# Dental Quacks, No. 3—Continued

**Published:** 1859-03

**Authors:** H. R. Smith


### DENTAL QUACKS, NO. 3—CONTINUED.

BY H. R. SMITH.

[The issue between Drs. Smith and Dills, of which the following embraces a portion, is a matter with which, so far as the personalities are concerned, the profession have little to do, and probably care quite as little. The principle involved, is one, however, that is of vital importance to the profession. Upon the rectitude of character, of the individual members of a profession, does the character of that profession depend. The principle which Dr. Smith sought to maintain in his first article, will be admitted by *all*, to be correct. The only point of issue being how *far* a man may go, without violating the principle. We think it is better to keep surely on the safe side, rather than to try to go as far as possible. Dr. D. is unfortunate in the matter,—his reply brings him prominent before the profession, as much as to say, I am the man to whom Dr. Smith refers; and then goes to work, to prove himself "not guilty" of the charge. There will be various opinions entertained, and it would have been better, had Dr. D. not appropriated any of Dr. S.'s article to himself at all.
We hope that this style of controversy will be less employed.—Ed.]

Messrs. Editors:—Circumstances compel me to come before you and the dental profession again in a manner that I would wished to have avoided. But when you open your pages to an assault on my private character, in reply to an article written on professional quackery, I feel obliged to appear again before the same readers, to explain what I have done and my motive for so doing.

In number one of the present volume of the *Register*, there was an article on Dental quackery, and to illustrate, "a case from life" was cited, every sentence of which, excepting names, was true as documents will prove.

In a week or two after the publication of the article, a gentleman (if the person could with impunity be called such) came into my office and with a very long visage opens the subject of the article. He owned up to the truth of it in full, but put in the flimsy excuse of its being the work of another, for which he was not responsible. He also took particular pains to visit the offices of several dentists in the city, and with great efforts abused me as far as his language could go—thinking thereby to exonorate himself by prejudicing them against me. But failing to accomplish his object to his mind, he seeks another channel.

We next find him in number two of the *Register*, over the name of Dr. C. C. D., with an extensive article—on what? Clearing up his character and proving my assertions false? No; none of these. What then? Why in his own estimation at least, he makes out a case of malicious persecution against an innocent and unoffending brother of the profession; and also proves his accomplice, the editor, out to be either an knave or fool.

But before we proceed further let me introduce to the profession the author of the article on Dental quackery, number two, in the December number of the *Register*.

The author is Dr. Cephas C. Dills, formerly of Piqua, Ohio. When the article was published he was a partner of a Dr. Hamilton, residing in New Castle, Indiana. He now, I am informed, resides in Peru, Indiana. His accomplice, the editor, is I. S. Drake, formerly of Xenia, Ohio, who has of late sported the titles of Doctor, Banker and Editor. He now edits a newspaper, at New Castle, Indiana.

It is not my intention to bandy words with Dr. Dills, or occupy much space in the *Register* in reviewing his very dignified article. Still, lest he thinks that I meant to slight his article, I would call the attention to a few things as we are passing.

In the first place I would call attention to his text, the last clause of which says that *naught will cure his wounded honor but my heart's blood*, &c.

Thus much if not sound dental ethics is at least very chivalric.

After much preliminary talk he says that my course was calculated "to stifle in the heart, what little aspirations he might have, to attain to the full stature of a man in the profession."

True, very true indeed. I would in fact stifle in infancy; aye, I would blight *in utero* all such deformities as his professional cause was *promising* to become. After some historical data he makes use of private correspondence to prove my maliciousness. But I think it will take a pretty strong imagination to draw any such conclusion. I shall let others judge of the tone indicated by these letters without any comment from me.

He next says that it is unnecessary for him to say "that the dental profession is in its infancy, &c." Well, to many of the profession, that will at least be news, for we are fain to believe that our profession has now grown to be an adult, and stands alongside on the same platform with her sister science, Medicine.

Dr. Dills spoke of his article in the newspaper as being a very good and smart joke—perhaps it is so. But like the joke perpetrated when the boys pelted the frogs—fun for the boys, but death to the frogs. So his joke may be fun for him, but death to the profession.

And lastly, he brings his accomplice in, to turn states evidence, who to clear Dr. Dills, is willing to acknowledge himself the scoundrel, thereby ending the suit.

But with all Dr. Dill's cunning, the readers of his article will undoubtedly recognize in him the counterpart of the animal who to avoid detection runs his head into a hole, but leaves his tail sticking out. I shall not sum up the evidence against the Doctor for it is too plain to be mistaken. I shall therefore leave him to swing on his own gallows, suspended by the cord as he himself adjusted it, hoping he will have a plesant time of it.

I regret exceedingly that with the good primary dental education Dr. Dills has had, with the examples set before him, he should so far swerve from the true professional path as to do as he has done. It also pains me to know that after he is detected in one wrong, he tries to exonorate himself by recklessly plunging into another; but, one that will serve to lower him in the estimation of all who read his article.

It may seem somewhat strange to some of the profession that I bring before the public a graduate of the college with which I had once the honor to be connected as one of the teachers.

As I had no private feelings to gratify, I did it with reluctance.

The evil did not originate with Dr. Dills, but was one that had been growing for some time, until with him it reached a culminating point.

It was not that I loved Dr. Dills less, but that I loved the College more; therefore, in hopes to save the parent from disgrace, I consented to the punishment of the undutiful child.

Now Messrs. Editors permit me to bear testimony that the course the Ohio College has ever taken and sustained, is to set the standard of excellence high, to frown upon and discountenance in every way and place, quackery and false pretensions, not only in the profession at large, but especially in all the students going out from the College. The Faculty and Trustees, through their President, have ever dismissed the graduates with the glorious injunction to be strictly honest in all their actions and pretensions, and to seek to honor their alma mater by keeping up the standard of the dental profession. And how much should it pain them when one of their graduates turns recreant to the noble principles taught by them.

And what can the feelings of such a one be, when he looks back to the night when the venerable President with full confidence in the receiver, gave him the diploma and Holy Writings, and at the same time called down the blessings of God to keep and protect him in a path of virtue and honesty.

Though not at present connected with the Ohio Dental College, I feel a warm friendship for her, because in her I see a moving power that is to qualify, regulate and protect the profession of which I feel proud to call myself a member. And from the professors of the College I have every reason to expect co-operation and sympathy in the stand I have taken in relation to quackery of the graduates, and when such is not the case, I shall feel that the cherished principles of the College are subverted and countenance given to quackery.

Am I to be sustained by an enlightened profession? or shall fear of offending the brazen-faced pretender seal the mouths and stop the pen. Time must tell.

